# Is cancer a pure growth curve or does it follow a kinetics of dynamical structural transformation?

**DOI:** 10.1186/s12885-017-3159-y

**Published:** 2017-03-07

**Authors:** Maraelys Morales González, Javier Antonio González Joa, Luis Enrique Bergues Cabrales, Ana Elisa Bergues Pupo, Baruch Schneider, Suleyman Kondakci, Héctor Manuel Camué Ciria, Juan Bory Reyes, Manuel Verdecia Jarque, Miguel Angel O’Farril Mateus, Tamara Rubio González, Soraida Candida Acosta Brooks, José Luis Hernández Cáceres, Gustavo Victoriano Sierra González

**Affiliations:** 1 Pharmacy Department, Oriente University, Natural Science Faculty, Patricio Lumumba Street, Santiago de Cuba, 90500 Cuba; 2 National Center of Seismology Research, Street 7 # 2 between L and M, Terraza, Santiago de Cuba, 90400 Cuba; 3 Research and Innovation Department, Oriente University, National Center of Applied Electromagnetism, Ave. Las Américas, Santiago de Cuba, 90400 Cuba; 4 Physics Department, Oriente University, Natural Sciences Faculty, Patricio Lumumba Street, Santiago de Cuba, 90500 Cuba; 5 Faculty of Sciences and Literature, Department of Mathematics, Izmir University of Economics, Izmir, 353300 Turkey; 6 Faculty of Engineering and Computer Sciences, Department of Computer Engineering, Izmir University of Economics, Izmir, 353300 Turkey; 7 ESIME-Zacatenco, Instituto Politécnico Nacional, México, DF 07738 Mexico; 8 Infantil Sur Hospital, Onco-Hematology Department, Santiago de Cuba, 90200 Cuba; 9 Conrado Benítez Oncological Hospital, Mastology Department, Santiago de Cuba, 90500 Cuba; 10 Dirección Municipal de Salud Pública, Servicio de Genética, Santiago de Cuba, 90500 Cuba; 11 Hospital Provincial Saturnino Lora, Servicio de Medicina Interna, Santiago de Cuba, 90500 Cuba; 12 Facultad de Ciencias Médicas “Diez de Octubre”, Universidad de Ciencias Médicas de La Habana, Avenida Independencia No. 8126, Esquina a Calle 100, Boyeros, La Habana, Cuba; 13 Grupo de las Industrias Biotecnológicas y Farmacéuticas (BioCubafarma), Havana, Cuba

**Keywords:** Fibrosarcoma Sa-37 tumor, Diffusion-controlled nucleation/growth mechanisms, Impingement mechanisms, Isothermal dynamical structural transformation

## Abstract

**Background:**

Unperturbed tumor growth kinetics is one of the more studied cancer topics; however, it is poorly understood. Mathematical modeling is a useful tool to elucidate new mechanisms involved in tumor growth kinetics, which can be relevant to understand cancer genesis and select the most suitable treatment.

**Methods:**

The classical Kolmogorov-Johnson-Mehl-Avrami as well as the modified Kolmogorov-Johnson-Mehl-Avrami models to describe unperturbed fibrosarcoma Sa-37 tumor growth are used and compared with the Gompertz modified and Logistic models. Viable tumor cells (1×10^5^) are inoculated to 28 BALB/c male mice.

**Results:**

Modified Gompertz, Logistic, Kolmogorov-Johnson-Mehl-Avrami classical and modified Kolmogorov-Johnson-Mehl-Avrami models fit well to the experimental data and agree with one another. A jump in the time behaviors of the instantaneous slopes of classical and modified Kolmogorov-Johnson-Mehl-Avrami models and high values of these instantaneous slopes at very early stages of tumor growth kinetics are observed.

**Conclusions:**

The modified Kolmogorov-Johnson-Mehl-Avrami equation can be used to describe unperturbed fibrosarcoma Sa-37 tumor growth. It reveals that diffusion-controlled nucleation/growth and impingement mechanisms are involved in tumor growth kinetics. On the other hand, tumor development kinetics reveals dynamical structural transformations rather than a pure growth curve. Tumor fractal property prevails during entire TGK.

## Background

Asymptotic growth indicates that a system shifts from positive feedback (which generates exponential growth) to negative feedback (which produces stabilizing growth). This shift is known as sigmoidal (“S-curve” or S-shaped growth). Systems that exhibit S-shaped growth-time behavior are characterized by constraints or limits to growth, as sickle cell disease [1], tumors [2], bacteria and microorganisms [3], among others. Other systems produce S-shaped transformation-time behavior, as crystals [4, 5].

Tumor growth kinetics (TGK) is not well understood so far. TGK has three well-defined stages: the first (Lag stage) is associated with the establishment of the tumor in the host. The second (Log or exponential stage) is related to rapid tumor growth. The third (Stationary stage) shows slow tumor growth asymptotically converging to a final volume [2]. It is expected a fourth stage (Death stage) of TGK, in which tumor dies because the nutrients are depleted by anorexia of animal or human host, showing a decline. This fourth stage is not considered in TGK due to ethical considerations [6, 7]. In mice, tumor burden should not usually exceed 10% of the host animal’s normal body weight [6].

During the last decades, tremendous efforts have been made by both experimentalists and theoreticians to search a suitable growth law for tumors, one of the most striking and interesting issues in cancer research [2, 8–11]. The Logistic equation has been used to describe TGK and the interactions among different competing populations with and without an external perturbation [12, 13]. The Logistic and von Bertalanffy equations have been reported to provide excellent fits for patients and mice bearing tumors, respectively [8]. In contrast, Marušic et al. [9] and Miklavčič et al. [11] show that the standard Gompertz model outperforms both Logistic and von Bertalanffy models. Marušic et al. [9] explain this disparity because the fit is dependent on the applied least squares fitting method. The Gompertz model is the most used to describe TGK [2, 8, 10, 11, 14].

The standard Logistic equation (Eq. 1) and the standard Gompertz equation (Eq. 2) are given by [8–11]:

1 $$ V(t)=\frac{K^{*}{V}_o{e}^{r^{*} t}}{K^{*}+{V}_o\left({e}^{r^{*} t}-1\right)} $$

2 $$ V(t)={V}_o{e}^{\left(\frac{\alpha}{\beta}\right)\left(1-{e}^{-\beta t}\right)} $$

where V(t) represents the untreated tumor volume at time t and V_o_ its initial volume at the beginning of observation (t = 0). Experimentally, V_o_ (reached in a time t_o_) is any tumor volume that satisfies the condition V_o_ ≥ V_meas_. V_meas_ is the minimum measurable tumor volume and reaches in a time, t_meas_. The constant r^*^ defines the growth rate and K^*^ is the carrying capacity [8, 12]. The parameter α is the intrinsic growth rate of the unperturbed tumor related to the initial mitosis rate. The parameter β is the growth deceleration factor related to the endogenous antiangiogenesis processes [11, 15] by an overexpression of different antiangiogenic molecules (i.e., Angiostatin, Thrombospondin-1 molecules) [15, 16]. As tumors are not perturbed with an external agent, this parameter β is not related to therapy-induced antiangiogenis [12]. Despite the interpretation of the parameter β, authors of this study believe that this parameter may be related to other endogenous antitumor processes, as cellular death processes (apoptosis, necrosis, metastasis and exfoliation) and interactions between tumor cells and immune cells [17]. Further experiments are required for a correct interpretation of this parameter.

An important part of tumor vital cycle has already happened long before V_meas_ is reached [17] and therefore it cannot be described with the Eqs. (1) and (2). However, this part of TGK may be fitted if an effective delay time (τ) is introduced in the Eqs. (1) and (2) [2, 18–20]. Besides, τ has been included in these two equations to describe Lag stage of bacteria- and microorganism growths [3]. τ has a crucial role in the modeling of biological processes [21]. The interesting question is if in the case with delay the Logistic model, named modified Logistic model (Eq. 3), or the Gompertz model, named modified Gompertz model (Eq. 4), is the best one for describing early tumor growth as it is believed in the case without delay (Eqs. 1 and 2). Equations (3) and (4) result of the substitution of t by (t-τ) in the Eqs. (1) and (2):

3 $$ V\left( t-\tau \right)=\frac{K^{*}{V}_{\tau}{e}^{r^{*}\left( t-\tau \right)}}{K^{*}+{V}_{\tau}\left({e}^{r^{*}\left( t-\tau \right)}-1\right)} $$

4 $$ V\left( t-\tau \right)={V}_{\tau}{e}^{\left(\frac{\alpha}{\beta}\right)\left(1-{e}^{-\beta \left( t-\tau \right)}\right)} $$

where V(t-τ) represents tumor volume at time (t-τ), meaning that the growth at present time t depends on the previous time (t-τ). Parameters τ and V_τ_ are the time and tumor volume corresponding to inflection point of TGK, respectively. Parameters r^*^, K^*^, α and β have been defined above in Eqs. 1 and 2.

Different findings have been documented in cancer, as: heterogeneity, anisotropy, fractal property, stiffness, surface roughening, curved surface, high macroscopic shear elastic modulus, among others [17, 21–25]. These findings have been also reported in crystals, despite noticeable differences between tumors and crystals, and in their growth mechanisms [26–30].

The classical Kolmogorov-Johnson-Mehl-Avrami model, named KJMA model (Eq. 5), and modified Kolmogorov-Johnson-Mehl-Avrami model, named mKJMA model (Eq. 6), have been used to fit entire sigmoidal curve of a crystal [26], given by

5 $$ p(t)=1-{e}^{-{(Kt)}^n} $$

6 $$ p(t)=1-{\left[1+\left(\lambda -1\right){(Kt)}^n\right]}^{-1/\left(\lambda -1\right)} $$

With

7 $$ K(T)={K}_o{e}^{-{E}_a/ RT}\;\left(\mathrm{Arrhenius}\ \mathrm{equation}\right) $$

where p(t) is the transformed fraction at t (fraction of grains that is transformed to crystal phase). n (n ≥ 0), K(T), λ (λ ≥ 1), K_o_, E_a_, R and T are the Avrami exponent, specific rate of transformation process that depends on temperature, impingement factor, the pre-exponential factor, effective (overall) activation energy of the transformation (or activation energy barrier to crystal formation), Boltzmann constant and temperature, respectively. RT represents the thermic kinetics energy. Arrhenius Eq. (7) is substituted in the Eqs. (5) and (6) to know K_o_ and E_a_.

In crystals, K is constant, proportional to the transforming volume/surface area and results of unbalanced diffusion processes (linked to heterogeneity). λ represents impingement mechanisms, as: capillarity effect, interfacial and superficial phenomena, among others. n is closely related to nucleation mechanisms, the existence of a lag stage, anisotropy, structural changes, vacancy annihilation, stiffness, surface roughening, curved surface, change of shape and high macroscopic shear elastic modulus of the forming and growing crystal. Additionally, n is inversely proportional to fractal dimension of the crystal. *n* ≥ 3 has been related to spherical shape of crystals, formation of micro-clusters of crystal seeds, high anisotropy and higher vacancies number [26–30].

On the other hand, nucleation and impingement mechanisms emerge to eliminate high energetic instabilities (by thermal fluctuation) during forming and growing crystal structure. Nucleation sites (or vacancy numbers) disorder the interior of forming and growing system and need be filled to guarantee their stability and growth. Deviation from integer value for n has been explained as simultaneous development of two (or more) types of crystals, or similar crystals from different types of nuclei (sporadic or instantaneous). Nucleation is either instantaneous, with nuclei appearing all at once early on in the process, or sporadic, with the number of nuclei increasing linearly with time [26–30].

KJMA and mKJMA models are phenomenological and not valid when T varies with time [31]. Furthermore, they are developed for the kinetics of phase changes to describe the rate of transformation of the matter from an old phase to a new one, taking into account that the new phase is nucleated by germ nuclei that already exists in the old phase. The Eq. (6) can be reduced to the Eq. (5) when λ tends to 1. Wang et al. [26] report that KJMA model cannot be applied to crystal growth when λ > 1 because there are phenomena (i.e., capillarity effects, vacancy annihilation, blocking due to anisotropic growth) that may cause violations to KJMA. Consequently, a misinterpretation of the kinetics may be given if these phenomena are ignored.

We are not aware that KJMA model and mKJMA model have been used to describe TGK. Nevertheless, in principle, these two models can be used to fit S-shaped growth of tumors, taking into account that “S-curve” is universal, the Eqs. (1, 2, 3, 4, 5 and 6) are phenomenological and the above-mentioned findings are common for both tumors and crystals. The application of the Eqs. (5) and (6) may reveal whether other findings not yet described are involved in TGK. Elucidating underlying mechanisms in entire TGK is of great importance for both understanding and planning antitumor therapies. The aim of this paper is to use, for the first time, KJMA and mKJMA models to describe the untreated fibrosarcoma Sa-37 TGK. Also, KJMA and mKJMA models are compared with modified Gompertz and Logistic models.

## Methods

### Mice

Twenty eight male (6–7 week, 18–20 g) BALB/c mice are studied. Animals are purchased from the National Center for Laboratory Animals Production (Havana, Cuba), housed in clear standard polycarbonate cages of 206 mm^2^ x 12 cm (4 animals/cage) with hard wood-shavings as bedding and given pellet BALB/c mice diet and tap water (sterilized and non-chemically treated) *ad libitum* under controlled environmental conditions, including a temperature of 23 ± 1 °C (Sattigungs thermometer of precision ± 1 °C, Germany), a relative humidity of 55 ± 5% (Fischer Polymeter of precision ± 1%, Germany), and a 12-h light/darkness cycle (lights on 7:00–19:00). Bedding and pellets are sterilized by autoclaving. They are changed daily. During the experiment the animals are firmly fixed on plastic boards and show uneasy and quick breathing during fixation. Survival checks for morbidity and mortality are made twice per day. Any animal found dead or moribund is subjected to gross necropsy.

### Tumor cell lines

Fibrosarcoma Sa-37 cell lines are received from the Center for Molecular Immunology (Havana, Cuba). Fibrosarcoma Sa-37 ascitic tumor cell suspensions, transplanted to the BALB/c mouse, are prepared from the ascitic form of the tumors. Subcutaneous tumors located in the right flank of the dorsolateral region of mice are initiated by the inoculation of 1x10^5^ viable tumor cells in 0.2 ml of 0.9% NaCl. The viability of the cells is determined by Trypan blue dye exclusion test and over 95%. Cell count is made using a hematocytometer. In cell count, a completely random distribution of fibrosarcoma Sa-37 tumor cells is observed without the presence of cellular clusters in the cellular suspension.

### Tumor growth kinetics

The period of study comprises the time interval from t = 0 (initial moment of tumor cells inoculation in the mice) up to tumor reaches a volume ≤ 1.5 cm^3^. Each individual tumor is observed to verify experimentally the minimum observable tumor volume, named V_obs_ (V_obs_ < V_meas_), reached at a time given, t_obs_ [2]. V_obs_ is observable but not measured. The volume of each individual tumor is calculated by means of the ellipsoid equation V = L_1_L_2_L_3_/6. L_1_, L_2_ and L_3_ (L_1_ > L_2_ > L_3_) are three perpendicular tumor diameters. Measurements of L_1_, L_2_ and L_3_ are made from tumor reaches V_meas_ up to 1.5 cm^3^. A vernier caliper with clamping screw (Model 530–104 of 0.05 mm of precision, Mitutoyo, Japan) is used. Each tumor diameter is measured three times for each individual tumor and then averaged, since its edge is not perfectly regular. This method permits tracking tumor development through the study with no need to slaughter the animals.

Mean doubling time (DT) is estimated for each individual tumor, once it reaches V_meas_. DT is the time required for a solid tumor to reach a twofold increase of its initial volume [17].

### Form factor and curvature radius of the tumor

**Fig. 1:**
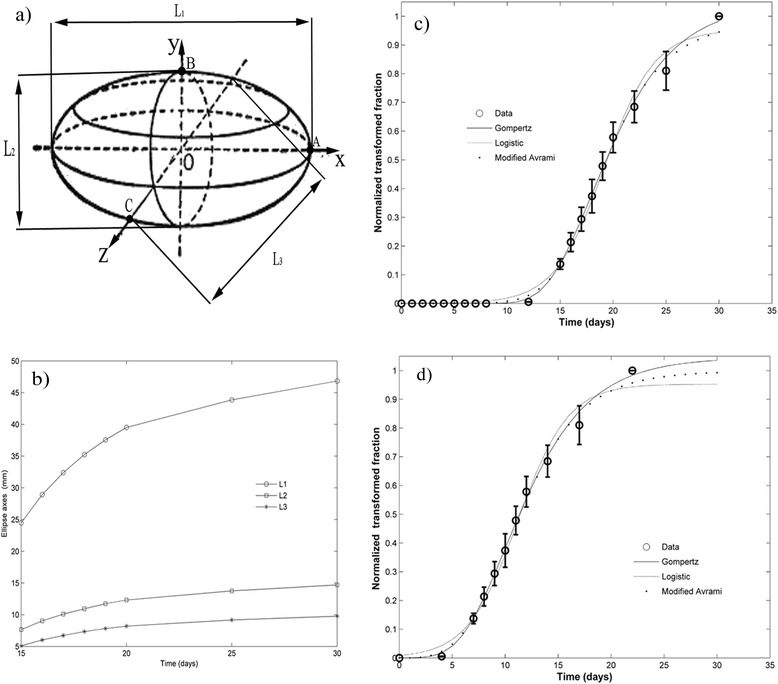
Fibrosarcoma Sa-37 tumor. **a** Schematic representation of its triaxial ellipsoid shape of L_1_, L_2_ and L_3_ diameters. **b** Time dependences of L_1_, L_2_ and L_3_. Experimental data (Mean ± standard error) of fibrosarcoma Sa-37 tumor normalized transformed fraction and growth curves fitted with modified models of Gompertz, Logistic and Kolmogorov-Johnson-Mehl-Avrami, from (**c**) t = 0 days and (**d**) t = 8 days

In order to know how tumor shape changes in time, form factor (FF, a measure of curved surface) and curvature radius (R_c_) are calculated in three perpendicular planes XY, XZ and YZ. Expressions to calculate FF and R_c_ are shown in Table 1. FF and R_c_ are calculated for each observation time. In each plane, R_c_ is calculated in the ellipse vertices (points where ellipse curvature is minimized or maximized), named R_c-L1_, R_c-L2_ and R_c-L3_ (see details in Table 1). FF and R_c_ may be also calculated via measuring all points of this closed quadric surface. In this case, the measurements of these points are tedious and require long time. L_1_, L_2_, L_3_ and planes XY, XZ and YZ are schematically depicted in Fig. 1a.

**Table 1:** Factor form and curvature radius by planes for the fibrosarcoma Sa-37 tumor

Planes	Form factor (FF)	Curvature Radius R_c_ (in mm)		
		R_c-L1_	R_c-L2_	R_c-L3_
xy	*πab*/2*p*_*ab*_^2^	*b* ^2^/*a*	*a* ^2^/*b*	-
xz	*πac*/2*p*_*ac*_^2^	*c* ^2^/*a*	-	*a* ^2^/*c*
yz	*πbc*/2*p*_*bc*_^2^	-	*c* ^2^/*b*	*b* ^2^/*c*

a (a = L_1_/2), b (b = L_2_/2) and c (c = L_3_/2) are the semi-axes of triaxial (or scalene) ellipsoid tumor on their respective planes. p_ab_, p_ac_ and p_bc_ are the ellipse perimeters on planes xy, xz and yz, respectively. R_c-L1_ is the curvature radius in the point A, R_c-L2_ in the point B and R_c-L3_ in the point C, as shown in Fig. 1a. It is important to point that the general expression for ellipse curvature radius on each plane is not given because the points of the closed curve do not experimentally measure

$$ {p}_{a b}=\pi \left( a+ b\right)\left[1+\frac{1}{4}{\left(\frac{a- b}{a+ b}\right)}^2+\frac{1}{64}{\left(\frac{a- b}{a+ b}\right)}^4+\frac{1}{256}{\left(\frac{a- b}{a+ b}\right)}^6\right] $$

$$ {p}_{a c}=\pi \left( a+ c\right)\left[1+\frac{1}{4}{\left(\frac{a- c}{a+ c}\right)}^2+\frac{1}{64}{\left(\frac{a- c}{a+ c}\right)}^4+\frac{1}{256}{\left(\frac{a- c}{a+ c}\right)}^6\right] $$

$$ {p}_{b c}=\pi \left( b+ c\right)\left[1+\frac{1}{4}{\left(\frac{b- c}{b+ c}\right)}^2+\frac{1}{64}{\left(\frac{b- c}{b+ c}\right)}^4+\frac{1}{256}{\left(\frac{b- c}{b+ c}\right)}^6\right] $$

### Model fitting

Equations (5) and (6) are used for the first time on the TGK. Below we describe the followed methodology.

First, the non-normalized experimental data are fitted with the Eqs. (3) and (4) from beginning of TGK (t = 0). τ and V_τ_ values are directly obtained in a plot of the first derivate of tumor volume versus tumor volume, named V’(t) versus V(t) plot [2]. In addition, TGK is fitted with the Eqs. (1) and (2) when the first point of the experimental data is V_obs_, V_oo_ (tumor volume reaches its diameter of 2 mm) or V_meas_, satisfying their specific initial conditions V(t = 0) = V_obs_, V(t = 0) = V_oo_ or V(t = 0) = V_meas_, respectively. These three initial conditions are valid if the respective co-ordinate origin is located at (t_obs_, V_obs_), (t_oo_, V_oo_) or (t_meas_, V_meas_). V_obs_ and V_oo_ are estimated from interpolation and extrapolation methods [2]. These analysis are shown in a V(t) versus t plot to compare the Eqs. (3) and (4), and the Eqs. (1) and (2), and also to know the values and estimation accuracies (or parameter error) of their parameters.

Second, as Eqs. (5) and (6) are normalized between 0 and 1, the experimental data is normalized by means of the normalization criterion p(t) = (V(t)-V_i_)/(V_f_-V_i_). V_i_ means the volume fraction of solid tumor at beginning of TGK or when the first point of TGK is V_obs_, V_oo_ or V_meas_. V_f_ represents the volume fraction of the solid tumor at the end of tumor growth. As V_i_ is very small (V_i_ tends to 0) this results in p(t) = V(t)/V_f_. Normalized experimental data are fitted with the Eqs. (1, 2, 3, 4, 5 and 6), in order to know the parameter values and their estimation accuracies for each equation, as well as to establish a comparison between them.

Third, different graphical strategies are followed, as: V(t-τ) versus t (for t ≥ 0); V(t) versus t (for t ≥ t_obs_); p(t) versus t (for t ≥ 0 and t ≥ t_obs_); ln(−ln(1-p(t))) versus ln(t) on a double-logarithmic plot obtained with the Eq. (5) (for t > 0); ln(−ln((1-p(t)^-(λ-1)^-1)/(λ-1))) versus ln(t) on a double-logarithmic plot obtained with the Eq. (6) (for t > 0); n_loc_ versus ln(t) and n_loc_ versus p(t) for both Eqs. (5) and (6), and t > 0. n_loc_ (n_loc_ ≥ 0) represents the instantaneous slope of these two equations at any given p(t). All these simulations are made from the mean values of n, λ, K and E_a_ obtained from fitting of normalized experimental data with the Eqs. (5) and (6). For the Eq. (5), n_loc_ is computed by means of ∂ln(1-p(t))/∂t. For the Eq. (6), n_loc_ is calculated by means of ∂ln((1-p(t)^-(λ-1)^-1)/(λ-1))/∂t. These graphical strategies are suggested by Wang et al. [26].

Fourth, n_loc_ is also estimated from the normalized experimental data, for KJMA and mKJMA models. For this, the definition of n_loc_, for each model, is applied to the normalized experimental data (p(t) versus t plot) when the first point of the experimental data is V_obs_.

The results of these last three points permit to know if the Eqs. (5) and (6) can be indistinctly used to describe TGK and to give a possible biophysics interpretation of their kinetic parameters.

### Criteria for model assessment

Since tumor growth is represented in biological research as series of volumetric measurements over time, we are presented with a classic case of least squares curve fitting. To fit an n-parameter nonlinear equation to tumor volume measurements, the Marquardt-Levenberg algorithm (an alternative to the Gauss-Newton algorithm) [14, 32] is used, which is the most widely used in nonlinear least squares fitting. Other algorithms have been used, as Nelder-Mead [33], which is not used because the standard deviation of the experimental data is small, even for a larger tumor.

As the Eqs. (3), (4) and (6) are overparameterized, the parameter estimation accuracy is also obtained from this algorithm. Also, as these three equations are multiparametric and the experimental data have associated error bars, it is important to point out that the error on the fit parameter is calculated multiplying the reported error on the fit parameters by the square root of the reduced chi-squared. For both non-normalized and normalized experimental data, the values and their estimation accuracies of the parameters for Eqs. (1, 2, 3, 4, 5 and 6), and five different fitting quality criteria: the sum of squares of errors, SSE (Eq. 8); standard error of the estimate, SE (Eq. 9); adjusted goodness-of-fit coefficient of multiple determination, *r*_*a*_^2^ (Eq. 10) predicted residual error sum of squares, PRESS (Eq. 11); and multiple predicted residual sum error of squares, MPRESS (Eq. 12) are computed from their individual values and used for model assessment (see details in [4]). These criteria are given by

8 $$ S S E={\displaystyle \sum_{j=1}^{n_1}{\left({\widehat{V}}_j^{*}-{V}_j^{*}\right)}^2} $$

9 $$ S E=\sqrt{\frac{{\displaystyle \sum_{j=1}^{n_1}{\left({\widehat{V}}_j^{*}-{V}_j^{*}\right)}^2}}{n_1- k}} $$

10 $$ {r}_a^2=1-\frac{n_1-1}{n_1- k}\;\left(1-{r}^2\right)=\frac{\left({n}_1-1\right)\kern0.5em {r}^2- k+1}{n_1- k} $$

11 $$ 1-{r}^2=\frac{{\displaystyle \sum_{j=1}^{n_1}{\left({\widehat{V}}_j^{*}-{V}_j^{*}\right)}^2}}{{\displaystyle \sum_{j=1}^{n_1}{\left({V}_j^{*}\right)}^2-\frac{1}{n_1}{\left({\displaystyle \sum_{j=1}^{n_1}{V}_j^{*}}\right)}^2}} $$

12 $$ PRESS=\frac{{\displaystyle \sum_{j=1}^{n_1-1}{\left[{\left({\widehat{V}}_j^{*}\right)}^{\acute{\mkern6mu}}-{V}_j^{*}\right]}^2}}{n_1- k} $$

13 $$ MPRESS(m)=\frac{{\displaystyle \sum_{j= m+1}^{n_1}{\left[{\left({\widehat{V}}_j^{*}\right)}^{\acute{\mkern6mu}}-{V}_j^{*}\right]}^2}}{n_1- m} $$

where *V*_j_^*^ is the *j*-th measured tumor volume at discrete time *t*_*j*_, j = 1, 2, …, n_1_, ${\widehat{V}}_j^{*}$ is the *j*-th estimated tumor volume by Gompertz, Logistic, KJMA or mKJMA model. *n*_1_ is the number of experimental points (n_1_ = 11). *k* is the number of parameters. *r* ^2^ and *r*_*a*_^2^ are goodness-of-fit and adjusted goodness-of-fit, respectively. The fitting is considered to be satisfactory when *r*_*a*_^2^ > 0.98. Higher *r*_*a*_^2^ means a better fit. (*V*_j_^*^)^*´*^ is the estimated value of *V*_j_^*^ when the model (Gompertz, Logistic, KJMA or mKJMA model) is obtained without the *j*-th observation. MPRESS removes the last *n*_1_ − *m* measurements. The model is fitted to the first m measured experimental points (m = 3, 4 or 5) and then from calculated model parameters the error between tumor volume estimates and measured values in the remaining *n*_1_ − *m* points is calculated. Least Sum of Squares of Errors is obtained when SSE is minimized in the Marquardt-Levenberg optimization algorithm.

### Comparisons between equations

The Eqs. (3) and (4) are compared when TGK begins at t = 0 days, taking as reference the Eq. (4). The Eqs. (1) and (2), and the Eqs. (2) and (4) are also compared when the first point of TGK is V_o_ (V_obs_, V_oo_ or V_meas_), being the Eq. (2) the reference. Furthermore, the Eqs. (5) and (6) are also compared when the first point of TGK is V_o_, using the Eq. (5) as reference. They are also compared with the Eq. (2) (when the first point of TGK is V_o_) or the Eq. (4) (when TGK begins at t = 0). Root Means Squares Errors (RMSE) and maximum distance (D_max_) values are used to compare these equations [2, 14], given by

14 $$ {D}_{\max }= \max \left|{F}_i-\left.{G}_i\right|\right. $$

15 $$ RMSE=\sqrt{{\displaystyle \sum_{i=1}^M\frac{{\left({F}_i-{G}_i\right)}^2}{M}}} $$

where *M* is the total number of points. *G*_*i*_ is the *i-th* calculated tumor volume with equation choice as reference (see above). *F*_*i*_ is the *i-th* calculated tumor volume by another equation compared.

A computer program is implemented in the MATLAB software (version R2011a, license number: 625596, San Jorge University, Zaragoza, Spain) to calculate the values of tumor volume, first derivate of tumor volume, and transformed fraction of tumor volume in each time. In addition, DT; FF; R_c_; RMSE; D_max_; SSE; SE; *r*_*a*_^2^; PRESS and MPRESS expressions are implemented in this program to calculate their values.

Each fit with the Eqs. (1, 2, 3, 4, 5 and 6) is performed for each animal’s growth curve, for both non-normalized and normalized data. The mean ± mean standard error of the parameters L_1_, L_2_, L_3_, tumor volume, first derivate of the tumor volume, r^*^, K^*^, α, β, FF, R_c_, τ, V_τ_, K, n, λ, E_a_, DT, estimation accuracy, RMSE, D_max_, SSE, SE, *r*_*a*_^2^, PRESS and MPRESS are calculated from their individual values. Mean standard error is calculated as (standard deviation)/ $\sqrt{N}$, where N is the total number of determinations. *N* = 3 is used for each average tumor diameter and *N* = 28 for the other parameters. Besides, this software permits performing curve fitting and to visualize the graphs of the graphical strategies above mentioned.

## Results

### Unperturbed fibrosarcoma Sa-37 tumor growth kinetics

The fibrosarcoma Sa-37 tumor exhibits a sigmoidal kinetics characteristic for both non-normalized and normalized experimental data. This S shape is observed when TGK begins at t = 0 (Fig. 1c) or the first point of TGK is V_obs_ (Fig. 1d) up to 1.5 cm^3^, which is reached at 30 days after tumor cells are transplanted into BALB/c mice. Vobs is observed in all tumors between 6 and 9 days. The higher relative frequency of V_obs_ is at t_obs_ = 8 days (24/28 = 85.7%). The Eq. (4) estimates V_obs_ in 0.000016 cm^3^ (0.031 cm in diameter) for t_obs_ = 8 days. This equation estimates V_oo_ (0.00416 cm^3^) at 9.8 days, in agreement with the experiment (around 10 days). V_meas_ (0.02 cm^3^) is observed between 10 and 12 days. The higher relative frequency of V_meas_ is at t_meas_ = 11 days (21/28 = 75%). The Eq. (4) estimates V_meas_ at t_meas_ = 10.8 days. From V_meas_, average DT estimated from non-normalized experimental data is 1.6 ± 0.4 days.

From V_obs_, both tumor and body temperatures remain practically unalterable (36.5 ± 0.1 °C) for each mouse. As tumor temperature is 36.5 °C (309.5 °K) and R = 8.3144 J/mol°K, RT = 2568.85 J/mol. Besides, surface roughening, compactness and stiffness of the fibrosarcoma Sa-37 tumor increase over time as its volume also increases, verified by both palpation and clinical observation.

Average values of L_1_, L_2_ and L_3_ values versus time are shown in Fig. 1b, corroborating that the tumor growth is anisotropic (prevails one preferential direction of growth, major diameter, named L_1_). In each mouse, shape changes of fibrosarcoma Sa-37 tumor are observed during entire TGK. Fibrosarcoma Sa-37 tumor grows spherically (L_1_ ≅ L_2_ ≅ L_3_) between 6 and 10 days after tumor cells are inoculated in BALB/c mice; then ellipsoidal with slightly irregular border and three different orthogonal well-defined axes (L_1_ > L_2_ > L_3_, from 11 up to 17 days); and lastly irregular-shaped, but three diameters of the tumor are still defined and measurable (from 18 up to 30 days). Seventeen days is the time that lapses so that solid tumor reaches 1 cm^3^. Complete loss of the fibrosarcoma Sa-37 tumor ellipsoidal shape (three diameters of the tumor are not well defined) starts from 30 days post-inoculation, as observed. This and ethical reasons [6] justify why the study period is up to 30 days.

The values of τ (15 ± 2 days) and V_τ_ (0.5 ± 0.05 cm^3^) are obtained from V’(t) versus V(t) plot (results not shown). The higher relative frequency of (15 days, 0.5 cm^3^) is observed for 57.1% (16/28) of tumors. As a result, in a first approximation, τ = 15 days and V_τ_ = 0.5 cm^3^ are introduced in the Eqs. (3) and (4) for the simulations.

### Parameters of each equation

Equations (1, 2, 3, 4, 5 and 6) fit well normalized data in each mouse and provided average values of their kinetic parameters, when TGK begins at t = 0 (Table 2 and Fig. 1c) or its first point is V_obs_ (Table 3 and Fig. 1d). For these equations, there is no problem with the convergence in the fitting of individual tumor growth data when the Marquardt-Levenberg optimization algorithm is used. This convergence is rapidly reached. The results are only shown for V_o_ = V_obs_ in order to know in depth the biggest part of Lag-phase. Comparisons of the Eqs. (1, 2, 3, 4, 5 and 6) are in agreement with small values of SE, SSE, PRESS and MPRESS (Tables 2 and 3), RMSE (≤0.001 cm^3^) and D_max_ (≤0.03 cm^3^). The mean value ± mean standard error of α, β, r^*^, K^*^, K, K_o_, n, λ and E_a_ parameters and the statistical criteria are given in Tables 2 and 3. The estimation accuracy of the parameters α, β, K^*^, r^*^, K, n and λ shown in Table 2 are 0.025 ± 0.001 days^−1^, 0.015 ± 0.001 days^−1^, 0.051 ± 0.002 days^−1^, 0.026 ± 0.002 days^−1^, 0.002 ± 0.001 days^−1^, 0.116 ± 0.056 and 0.577 ± 0.041, respectively. The estimation accuracy for these respective parameters shown in Table 3 are 0.030 ± 0.002 days^−1^, 0.021 ± 0.002 days^−1^, 0.070 ± 0.005 days^−1^, 0.030 ± 0.004 days^−1^, 0.008 ± 0.002 days^−1^, 0.481 ± 0.022 and 0.444 ± 0.014.

**Table 2:** Mean ± mean standard error of the parameters and criteria for model assessment using in fitting of fibrosarcoma Sa-37 tumor growth data with modified models of Gompertz (Eq. 3), Logistic (Eq. 4) and Kolmogorov-Johnson-Mehl-Avrami (mKJMA) (Eq. 6) from t ≥ 0 days

	Modified models on normalized data		
	Gompertz	Logistic	mKJMA
X_1_ ± SD	(0.473 ± 0.053) days^−1^	(0.953 ± 0.031) cm^3^	(0.052 ± 0.003) days^−1^
*X*_2_ ± SD	(0.233 ± 0.031) days^−1^	(0.425 ± 0.043) days^−1^	7.599 ± 1.247
X_3_ ± SD	-	-	2.271 ± 0.521
r^2^ ± SD	0.995 ± 0.004	0.989 ± 0.007	0.994 ± 0.004
*r*_*a*_^2^ ± SD	0.995 ± 0.004	0.989 ± 0.007	0.994 ± 0.004
SE ± SD	0.022 ± 0.007	0.032 ± 0.009	0.023 ± 0.007
SSE ± SD	0.009 ± 0.006	0.019 ± 0.0115	0.010 ± 0.006
PRESS ± SD	0.0004 ± 0.0003	0.0008 ± 0.0004	0.0004 ± 0.0002
MPRESS1 ± SD	0.0006 ± 0.0004	0.0012 ± 0.0007	0.0006 ± 0.0004
MPRESS2 ± SD	0.0006 ± 0.0004	0.0013 ± 0.0007	0.0007 ± 0.0004
MPRESS3 ± SD	0.0007 ± 0.0004	0.0014 ± 0.0007	0.0007 ± 0.0004
K_o_ (days^−1^)	-	-	0.056 ± 0.004
E_a_ (J/mol)	-	-	187.331 ± 157.609
RT (J/mol)	-	-	2 568.85

X_1_ and *X*_2_ variables signify the parameters α and β in the modified Gompertz model whereas these two variables symbolize the parameters K^*^ and r^*^ in the modified Logistic model, respectively. X_1_ *X*_2_ and X_3_ represent K, n and λ in mKJMA model, respectively. RT is the thermal energy calculated. T, K_o_, E_a_, SE, SSE, *r*_*a*_^2^, PRESS, MPRESS and SD are the temperature, pre-exponential factor, activation energy (activation enthalpy) of the transformation, standard error of the estimate, sum of squares of errors, adjusted *r* ^2^, predicted residual error sum of squares, multiple predicted residual sum error of squares and standard deviation, respectively. *r* ^2^ is the goodness-of-fit. Details of SE, SSE, *r* ^2^, *r*_*a*_^2^, PRESS and MPRESS are given in [2, 14]

**Table 3:** Mean ± mean standard error of the parameters and criteria for model assessment using in fitting of fibrosarcoma Sa-37 tumor growth data with modified models of Gompertz (Eq. 2), Logistic (Eq. 1) and Kolmogorov-Johnson-Mehl-Avrami (mKJMA) (Eq. 6) from t ≥ 8 days

	Modified equations on normalized data		
	Gompertz	Logistic	mKJMA
X_1_ ± SD	(0.473 ± 0.053) days^−1^	0.953 ± 0.031	(0.083 ± 0.008) days^−1^
*X*_2_ ± SD	(0.233 ± 0.031) days^−1^	(0.425 ± 0.043) days^−1^	3.409 ± 0.529
X_3_ ± SD	-	-	1.509 ± 0.366
r^2^ ± SD	0.991 ± 0.005	0.982 ± 0.011	0.991 ± 0.006
*r*_*a*_^2^ ± SD	0.990 ± 0.007	0.979 ± 0.013	0.990 ± 0.007
SE ± SD	0.030 ± 0.009	0.044 ± 0.013	0.030 ± 0.009
SSE ± SD	0.009 ± 0.006	0.019 ± 0.010	0.009 ± 0.006
PRESS ± SD	0.0008 ± 0.0005	0.0015 ± 0.0008	0.0007 ± 0.0005
MPRESS1 ± SD	0.0011 ± 0.0007	0.0022 ± 0.0014	0.0010 ± 0.0007
MPRESS2 ± SD	0.0012 ± 0.0008	0.0024 ± 0.0016	0.0012 ± 0.0007
MPRESS3 ± SD	0.0014 ± 0.0009	0.0026 ± 0.0018	0.0013 ± 0.0009
K_o_ (days^−1^)	-	-	0.109 ± 0.014
E_a_ (J/mol)	-	-	706.97 ± 393.15
RT (J/mol)	-	-	2 568.85

X_1_ and *X*_2_ variables signify the parameters α and β in the modified Gompertz equation whereas these two variables symbolize the parameters K^*^ and r^*^ in the modified Logistic equation, respectively. X_1_ *X*_2_ and X_3_ represent K, n and λ in Modified KJMA equation, respectively. RT is the thermal energy calculated. SE: Standard error of the estimate. *SSE* sum of squares of errors. *r*_*a*_^2^: adjusted r^2^. *PRESS* Predicted residual error sum of squares and *MPRESS* Multiple predicted residual sum error of squares. *SD* Standard deviation. K_o_ is the pre-exponential factor. E_a_ is the activation energy (activation enthalpy) of tumor cell nucleation. *r* ^2^ is the goodness-of-fit. Details of SE, SSE, *r* ^2^, *r*_*a*_^2^, PRESS and MPRESS are given in [2, 14]

Although the results of the fitting of the experimental data with Eq. (5) are not shown in Tables 2 and 3, it can be verified that K = 0.0758 days^−1^ and n = 2.7503. Estimation accuracies of K and n are 0.004 ± 0.002 days^−1^ and 0.321 ± 0.087, respectively. On the other hand, average DT of 1.7 ± 0.2 days is obtained with Eq. (2). Average DT = 0.9 ± 0.3 days is predicted with Eq. (6). As expected, these DT values are indistinctly obtained from non-normalized and normalized data.

Tables 2 and 3 show that parameters α, β, K^*^ and r^*^ have equal values. α and β values differ from those reported by Cabrales et al. [2] in 0.04 and 0.033 days^−1^, respectively. Values for α and r^*^ differ in 0.048 days^−1^, indicating that α ≅ r^*^. In addition, Tables 2 and 3 evidence that K values are one order smaller than α and r^*^ values, and the values of E_a_ are smaller than RT. K_o_, K and E_a_ values shown in the Table 3 are higher than those in Table 2. Values for n and λ shown in Table 3 are smaller than those in the Table 2. Although the results are not shown, it can be verified that K_o_, K and E_a_ values increase, and n and λ values decrease with respect to those shown in Table 3 when tumor volume increases regarding to V_obs_.

On the other hand, it can be verified that results shown in Table 3 coincide with those obtained from fitting of no-normalized data with Eqs. (1, 2, 3 and 4), when the first point of experimental data is V_obs_, V_oo_ or V_meas_. Nevertheless, when TGK begins for a tumor volume higher than V_meas_, α, β, K^*^ and r^*^ change compared with those shown in Table 3 (results not shown). In addition, Eqs. (3) and (4), and Eqs. (1) and (2) fit well to no-normalized data in each mouse when TGK beginning at t = 0 and the first point is V_obs_, V_oo_ or V_meas_.

**Fig. 2:**
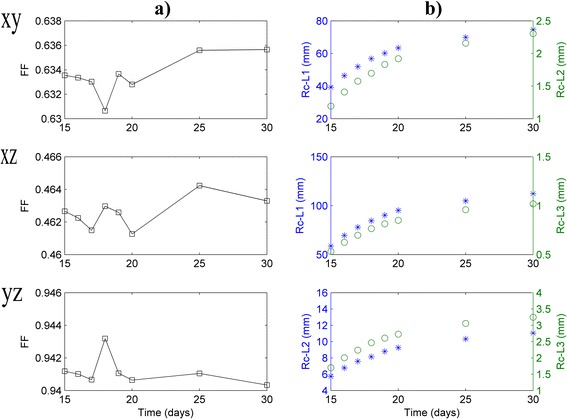
Shape change of fibrosarcoma Sa-37 tumor. **a** Tumor factor form (FF) versus time. **b** Tumor curvature radius versus time on points A, B and C, given by R_c-L1_, R_c-L2_ and R_c-L3_, respectively. FF, R_c-L1_, R_c-L2_ and R_c-L3_ are given on three perpendicular planes xy, xz and yz. These *curves* are shown for t ≥ t_obs_

Figure 2 shows that FF and R_c_ depend on time and the plane XY, XZ or YZ. The higher values of FF and R_c_ are observed in plane YZ and L_1_ diameter (along axis x), respectively. Moreover, this figure reveals that R_c-L1_, R_c-L2_ and R_c-L3_ increase with time, being R_c-L1_ > R_c-L2_ > R_c-L3_.

The graphical strategies for constant temperature show similar behaviors to those shown in [26] and therefore, they are not shown in this study. Nevertheless, it can be verified that simulations of ln(−ln(1-p(t))) versus ln(t) plot and ln(−ln((1-p(t)^-(λ-1)^-1)/(λ-1))) plot exhibit linear and non-linear increases, respectively. n_loc_ versus p(t) plot shows that n_loc_ remain constant for KJMA model, whereas n_loc_ non-linearly decreases as p(t) increases, for mKJMA model. This non-linearity is noticeable when λ increases. In addition, simulation of n_loc_ versus ln(t) plot for Eq. (5) predicts a linear behavior of n_loc_ in the time. However, this plot for Eq. (6) evidences that n_loc_ drops exponentially in the time (continue and smooth curve). This deviation from linearity starts at the very early stages of the entire TGK, when λ > 1, being noticeable when λ increases.

**Fig. 3:**
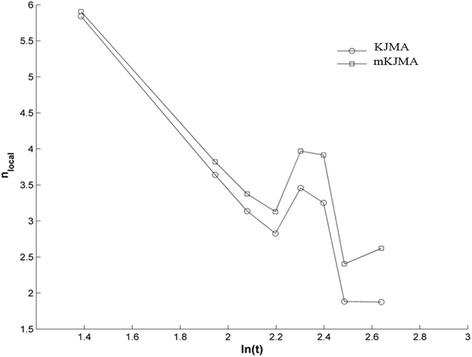
n_loc_ versus ln(t) plot on the normalized experimental data for KJMA and mKJMA models, and t ≥ t_obs_

The analysis of n_loc_ versus ln(t) plot on the normalized experimental data reveals that n_loc_ drops with time showing a jump (around 10 days) for both KJMA and mKJMA models, as it can be seen in Fig. 3. It is important to point out that this jump coincides with the shift in the fibrosarcoma Sa-37 tumor from spherical to ellipsoidal shape. Obtained values for n_loc_ with mKJMA are higher than those for the KJMA model. Besides, for both models, n_loc_ > 4 (before 6 days) and 3 ≤ n_loc_ ≤ 4 (between 6 and 10 days) are observed.

## Discussion

The results of this study are valid for the unperturbed fibrosarcoma Sa-37 tumor, experimentally transplanted to BALB/c mice. As shown, parameter n_loc_ is a better descriptor than n for the entire TGK. The plausibility of V(t) versus t plot and/or p(t) versus t plot for TGK analysis is also suggested, in agreement with [34]. Equations (1, 2, 3, 4, 5 and 6) can be used to fit normalized experimental data from Sa-37 tumor, as assessed by the high *r*_*a*_^2^ values, low values of SSE, SE, PRESS, MPRESS as well as overall estimation accuracy. Each equation has high prediction capability and good missing data handling. This further supports sigmoid laws universality [3, 35].

Despite mentioned in the previous paragraph, a weighted least square minimization in formula (6) may be proposed for selection of the best model, taking into account the uncertainty of the individual measurements of the tumor volume and the fact that the larger the volume, the larger the standard deviation. This and other statistical criteria [33] in tumor volumes with smaller and larger standard deviations will be included in a further study.

As obtained, V_o_ can be indistinctly chosen as V_obs_, V_oo_ or V_meas_ since Eq. (2) behaves similarly when any of them is used in experimental data fitting. Unlike Eqs. (2) and (4), the parameters of Eq. (6) depend on the first point of TGK, indicating that it senses the microstructural changes from beginning of TGK (t = 0).

The good fits yielded by Eqs. (1) and (3) are in contrast with [8, 9, 11, 33]. This can be due to the omission of larger tumors, since mice were slaughtered earlier, following [6]. That is why, p(t) and n_loc_ do not reach the values of 1 and 0, respectively. In crystals, p(t) = 1 and n_loc_ = 0 [26].

Equation (5) should not be used for TGK interpretation, since λ > 1; its parameters differ respect to those of Eq. (6) (Tables 2 and 3, and Fig. 3) and graphical strategies are noticeably different for these two equations. This agrees with Wang et al. [26]. Accordingly, results obtained with Eq. (5) have not been exposed here.

The close relationship between fibrosarcoma Sa-37 tumor spherical shape and n_loc_ ≥ 3 is corroborated in this study. Similar finding is reported in crystals [28–30]. This tumor spherical shape may be vital for tumor growth due to a lower surface curvature, in agreement with [2, 36–38]. Jump of n_loc_ and the change from spherical to non-spherical shape may be related to a shift from avascular (before 10 days) to vascular growth phase (after 11 days). Transition between these two phases has been previously reported [36, 37]. The observed n_loc_ jump corresponds to a transition of high (before n_loc_ jump) to low (after n_loc_ jump) value of n_loc_, suggesting the occurrence on TGK of two types of growth mechanisms that happen at different time scales: nucleation (below 10 days) and pure growth (above 11 days). Nucleation is expected at vascular growth phase, mainly at its very early stages, by high values of n_loc_ and it is the stochastic stage of a forming and growing system. This later may be due to the Brownian motion (a fractal stochastic process) of thermally fluctuating and energetically unstable tumor cells in suspension at t = 0.

High energetic instabilities at avascular growth phase are mitigated by nucleation mechanisms, suggesting a high micro-anisotropy, confirmed by n_loc_ ≥ 5. Micro-anisotropy leads to random formation of non-uniform and energetically unstable cellular micro-clusters, which establish a space-time competence for nutrients, oxygen and energy, resulting in high micro-heterogeneities, as reported in multicellular spheroid models [36–38]. This may explain the existence of the entropy production [39] and the diffusion limited aggregation at avascular tumor growth (mainly at its very early stages of TGK) because the tumor cells move randomly in Brownian motion, forming fractal clusters when diffusion is the main transport mechanism. Brownian motion and diffusion limited aggregation are stochastic rather than deterministic processes with random fractal dynamics. This diffusion limited aggregation may have an impact in TGK [40] and result in tumor cells packed in a multicellular spheroid not yet connected to the host’s blood supply, in agreement with [36–39, 41].

The formation of these cellular micro-clusters discards the occurrence of a burst nucleation, which means that all nucleation sites are immediately saturated at t = 0. Burst nucleation is reached for K → ∞, λ = 1, n → ∞ and/or DT → 0, in contrast with the results of this paper and with duration of Lag stage of TGK observed in preclinical (several days) and in clinical (several months and years) studies. Additionally, the existence of cellular micro-clusters may suggest that a tumor solid seed (or smallest size of a solid tumor), long before of V_obs_, may be essentially formed via heterogeneous nucleation mechanisms, as previously hypothesized Cabrales et al. [2]. This via is confirmed in this study by non-integer values of n and n_loc_, as in crystals [28–30].

Nucleation mechanisms may help to form these cellular micro-clusters by filling the high nucleation sites (or vacancies), which may correspond with unoccupied sites of the cancer cells. The existence of these sites may be justified because n_loc_ ≥ 5; this can lead to a higher number of heterogeneous sites, making unstable both the forming cellular system and the cellular micro-clusters. This process may be stabilized and ordered by both inter-cellular interactions and the overlapping of diffusion fields of tumor cells, a matter that agrees with [19, 36, 41], suggesting the existence of soft impingement mechanisms during the avascular growth phase. These mechanisms are also confirmed because λ > 2, as in crystals [26–28]. Nucleation and soft impingement mechanisms may explain, in part, why a slightly better binding of cancer cells with less detachment, in agreement with [42].

The filling of vacancies may explain why n_loc_ drops up to the jump of n_loc_. After n_loc_ jump, n_loc_ increases probably because pure growth mechanisms emerge and prevail over nucleation mechanisms. If pure growth mechanisms do not emerge, nucleation sites are completely saturated (n_loc_ tends to 0) in less than 30 days, in contrast with the results shown in Fig. 3. It should be expected that n_loc_ tends to 0 for larger tumors (≥3 cm^3^, which is reached long past 30 days) because TGK decelerates at stationary stage of TGK (cell-production-to-cell-loss rate is very slow or unalterable). This ratifies that TGK cannot be linear nor exponential (the host cannot fully sustain solid tumors due to their sizes would be bigger than host size). Accordingly, solid tumors are cooperative boundless systems, in agreement with the S-shape of tumor growth, and the fact that Eq. (6) has to level off at both extremes to represent almost no binding at the beginning of TGK and saturated binding at the final of TGK.

Heterogeneity and anisotropy of the fibrosarcoma Sa-37 tumor at vascular growth phase are confirmed by palpation; time changes of n_loc_, L_1_, L_2_, L_3_, FF and R_c_; irregular border, deformation and surface roughening [2, 17, 25] and are associated with compactness, stiffness and surface tension of the tumor [23, 24, 43]. Anisotropy produces preferred directions of growth, minimizing surface tension.

Brownian motion and cellular micro-clusters at very early stages of TGK; Figs. 1, 2 and 3; and the irregular border, surface roughening and stiffness of the tumor at vascular growth phase may suggest that forming, growing and transforming cellular system along TGK happens in a fractal space-time; as a consequence the fractional Hausdorff dimension (D_H_) is higher than the topologic dimension (D_T_), as it corresponds to a fractal space [44]. This means that although D_T_ = 0 for tumor cells in suspension (considered as a set of points) at t = 0, 0 < D_H_ < 1. It is expected that the forming and growing cellular system on TGK pass through different spatial patterns, starting from worm-like linear structures (D_T_ = 1 and 1 < D_H_ < 2); then, fish-like plane structures (D_T_ = 2 and 2 < D_H_ < 3); spatial solid-like structures (D_T_ = 3 and 3 < D_H_ < 4); and lastly, space-time structure (D_T_ = 4 and 4 < D_H_ < 5 or higher dimensions). It is possible that these two later structure types are reached once the tumor solid seed and vascular growth phase are formed, respectively. This is in contrast with [45] and agrees with [46]. Waliszewski and Konarski [45] obtain that the value of the mean temporal fractal dimension decreases along the curve approaching integer value because the fractal structure is lost with tumor progression. Shim et al. [46] correlate the S-shaped time increase of tumor fractal dimension, with textural parameters (i.e., hardness) and the growth in the time of space-time branching structures (or patterns). These structures are linked to the abnormal network of blood vessels, in agreement with the findings of the present study. This and the inverse relation between p(t) and n_loc_ (Figs. 1, 2 and 3) may suggest that n_loc_ and the tumor fractal dimension are inversely related, as it takes place in crystals [30]. Time changes in D_T_ and D_H_ may explain, in part, why tumor cells in vitro form colonies and grow in layers, unlike the normal cells, which do not form colonies [47].

Fractal properties of tumors have been correlated with its microstructure, microscopic coherent local deformation processes (or local dynamical rearrangements), mitosis rate, heterogeneity, anisotropy, complexity degree, spatial-temporal coherence, self-organization, self-stabilization, self-symmetry, self-ordering, self-similarity, mechanical properties (stiffness, compactness and surface roughening), temporal changes of nontrivial shape and dynamical structural intrinsic transformations [21, 35, 48–51].

Tumor fractal dimension may suggest that the tumor is a type of fractal, named space-filling fractal that continuously attempts to fill in the area leaving no empty holes. The space-filling pattern is formed by placing some non-overlapping units of smaller sizes. This may confirm the existence of annihilation of vacancies, in agreement with Molski and Konarski [48], confirming that solid phase of TGK is spatially coherent and therefore, all tumor cells co-operate collectively producing spatial-temporal organization and complex patterns.

The above discussed suggests that TGK is a fractal from its beginning (t = 0), unprecedented in the literature. This statement agrees with [52], in which is demonstrated the fractal origin of the Gompertz equation. Izquierdo-Kulich et al. [52] explain their results because α and β are connected with morphology of the tumors, specifically with the fractal character of them.

Small values of E_a_ and minimal tumor surface tension [23] may explain time evolution of V(t), p(t), n_loc_, D_T_/D_H_, shapes of the fibrosarcoma Sa-37 tumor. In addition, small values of E_a_ corroborate that vacancies require small amount of energy for their creations. They have received insufficient attention and may have an important role in the carcinogenesis, production/lost rate of tumor cells, and in mechanics [23, 24] and dielectric [53] properties of a forming and growing cellular system, during entire TGK. This may be explained because vacancies disorder these cellular systems, leading to structural, morphological and electrical instabilities [21, 48, 49, 51]. As a result, impingement mechanisms emerge during TGK in order to guarantee the most efficient space-filling and to stabilize the forming and growing cellular system, in order to maximize its exchanges of nutrients in the minimum amount of space and therefore to maximize the tumor growth and its stabilization. After n_loc_ jump, soft impingement mechanisms guarantee the growth, stabilization and survival of the tumor by branching structure (abnormal vascular network) [14–17], the different abnormal signaling pathways, the interactions that happen in the tumor and/or other uncontrolled environmental factors [17, 23, 36, 54, 55]. As a result, the tumor cells do not multiply in an unregulated manner, as reported in [17], but they are regulated by the number of vacancies available to be filled. Furthermore, intrinsic local dislocations lead to dynamical rearrangements of tumor cells, suggesting that dynamical structural intrinsic transformations take place along the entire TGK. This indicates that the forming and growing cellular system passes through different dynamical conformational states or meta-stable configurations. These configurations are rearrangements of the cancer cells that take place over a wide energy range due to the large number of stabilized and ordered cellular configurations. This agrees with Guha [56], who reports that a change of state takes place if there is an unbalanced force anywhere within the system, or between the system and its surrounding, leading to variations in pressure or elastic stress which give rise to the tumor expansion. This brings about that TGK may be limited and controlled by vacancies, which are governed by nucleation/growth and impingement mechanisms, and dynamical structural intrinsic transformations. As a result, cancer self-renews constantly and TGK is a highly coordinated dynamic multi-step process, in agreement with [57].

On the other hand, these dynamical structural intrinsic transformations may explain, in part, immune resistance mechanisms and low effectiveness of some antitumor therapies (i.e., immunotherapy when solely applied), in agreement with [58]. This may be explained because these transformations may be responsible for structural and stereochemical changes on membrane-bound receptor-ligand immune checkpoints that promote the tumor activity. As a result, ligand-receptor interactions are perturbed due to the expression of immune-checkpoint proteins which are disregulated [58].

The results may evidence that entire TGK is not only due to imbalance between cell production and cell loss [17] and other hallmarks of cancer [17, 54, 55], but also to diffusion-controlled nucleation/growth and impingement mechanisms, and dynamical structural intrinsic transformations, which may be the key to understand how a solid tumor arises and grows. These findings are often ignored in literature and may indicate that TGK is about dynamical structural transformations, instead of pure growth kinetics. They may explain why K is an order smaller than α/r^*^; DT value estimated with the Eq. (6) is smaller than that estimated experimentally and with the Eq. (2); the differences between the values of K, n, λ and E_a_ report in the Tables 2 and 3; and the difference of n_loc_ versus ln(t) for KJMA and mKJMA models (Fig. 3). On the other hand, if these findings are not considered on entire TGK, then pure growth mechanisms prevail in it, meaning that K ≅ α ≅ r^*^ and DT estimated experimentally and with Gompertz, Logistic and mKJMA models are equals, in contrast with results here shown.

Besides, the prevalence of these findings at avascular growth phase ratify that an important part of vital cycle of a solid tumor occur before it is clinically detected, in agreement with [17]. On the other hand, the Eq. (6) senses the microstructural changes that happen during the entire TGK, mainly at avascular growth phase, in contrast to Eqs. (1, 2, 3 and 4).

Many questions may arise, as: how n, n_loc_, λ and E_a_ depend on α, β and DT, which characterize the histological characteristics of a solid tumor? How an immune-deficient or immune-competent organism affect n, n_loc_, λ and E_a_ values? Can the Eq. (6) be modified to fit the perturbed tumor growth kinetics with an external agent? among others. This first study cannot give answers to all these questions. Relevant biological and clinical data may now be gathered in a systematic manner in order to test our theory or any other quantitative model derived using a methodology similar to ours, with the aim of helping to understand, and potentially handling, the process of tumor growth. Future studies will provide in-depth experimental findings that permit a best interpretation of the parameters of mKJMA model in cancer.

## Conclusions

In conclusion, modified Kolmogorov-Johnson-Mehl-Avrami is adequate to describe unperturbed transplanted fibrosarcoma Sa-37 tumor growth, which is not a purely growth kinetics, but kinetics of dynamical structural intrinsic transformation, involving diffusion-controlled nucleation/growth and impingement mechanisms. Besides, TGK follows a fractal nucleation and growth model.

## Abbreviations

λ: Impingement factor
α: The intrinsic growth rate of the tumor
τ: The time that corresponds to inflection point of TGK
*r*_*a*_^2^: Adjusted goodness-of-fit coefficient of multiple determination
D_H_: Hausdorff dimension
D_max_: Maximum distance
D_T_: Topologic dimension
DT: Tumor doubling time
E_a_: Effective (overall) activation energy of the transformation
FF: Tumor form factor
K^*^: The carrying capacity
K: Specific rate of transformation process
KJMA: Kolmogorov-Johnson-Mehl-Avrami classical model
K_o_: The pre-exponential factor
mKJMA: Modified Kolmogorov-Johnson-Mehl-Avrami model
MPRESS: Multiple predicted residual sum error of squares
n: The Avrami exponent
p(t): The transformed tumor fraction at t
PRESS: Predicted residual error sum of squares
r^*^: The growth rate
R: Boltzmann constant
R_c_: Tumor curvature radius
RMSE: Root Means Squares Errors
RT: Represents the thermic kinetics energy
RT: The thermic kinetics energy
SE: Standard error of the estimate
SSE: The sum of squares of errors
T: Temperature
TGK: Tumor growth kinetics
V_τ_: Tumor volume corresponding to inflection point of TGK
V(t-τ): Tumor volume at time (t-τ)
V_meas_: Minimum measurable tumor volume
V_o_: Initial tumor volume
V_obs_: Minimum observable tumor volume
V_oo_: Tumor volume reaches a diameter of 2 mm
β: The growth deceleration factor related to the endogenous antiangiogenesis processes
